# Incidents of Animal Life

**Published:** 1886-11-13

**Authors:** 


### Incidents of Animal Life.

Collated by the Rev. F. O. MORRIS, B.A.,
Rector of Nunburnholme, Yorkshire; author of "British Birds," etc.

" He prayeth best, who loveth best
All things both great and small;
For the dear God who loveth us,
He made and loveth all."—Coleridge.

**A Loyal Husband.**

The following story is told of George Stephenson—the father of railways—showing how loving and tender a heart he kept through his busy life. He went to stay for a short time at his house—Alton Grange; and one day he noticed a robin fluttering outside the window, and beating its wings against the panes, as if eager to get in. He went upstairs, and there found in an unused room a robin's nest, with one of the parent birds sitting over the young ones—but all, alas! dead. The poor little bird outside still beat against the panes, and on the window being opened it flew into the room and dropped upon the floor exhausted. Mr. Stephenson took it up tenderly, carried it downstairs, and had it warmed and fed. It revived for a time, and while it lived was one of his pets, for he always loved animals. Unable, however, to recover from all its sufferings during the three days it was shut out of the room, it soon died. The room had been unused for some time: and the window being open the robins had flown in and built their nest within. The servant afterwards accidentally closed the window, and thus the one inside was starved to death, yet would not leave its nest; and the poor little bird outside had tried to bring relief, and died in consequence of its sufferings. We may learn a lesson both from the man and the bird.

**A Goat Story.**

A gentleman, who had taken an active part in the Scotch rebellion of 1745, escaped after the battle of Preston Pans to the West Highlands, where he was received by a relative of his, a lady, who being, like himself, attached to the Stuarts, was glad to offer him an asylum. But as the search was very strict after the rebels, who, if taken, were executed, it was necessary for him to be concealed where there would be no danger of discovery. He was therefore conducted by one of the lady's servants to a cave in a sequestered part of the mountains, and being furnished with a supply of food for some little time, was left to take care of himself. The only way of entering his retreat was by a small opening, through which he had to creep, carrying his provisions with him, and feeling his way with one hand at every step. A little way from the mouth, the cave became more lofty, and still advancing cautiously in the darkness, he presently became aware of something that stopped his further progress. Unable to see what it was, and afraid to strike anything in the dark with his dirk, he stopped, and felt carefully around for the object, and soon perceived that it was a goat with a kid. This would not have been an unpleasant discovery had he not feared that the owner, following the goat hither, might betray him to his enemies: otherwise she might supply him with nourishment. Soon, however, he discovered that she was in great pain, and then, carefully feeling about her, perceived that one of her legs was broken. Fortunately he was not without some knowledge of the treatment of animals; he therefore bound up her leg with his garter, and offered her bread to eat. But her mouth was parched, and she refused the bread; he then gave her water, which she drank eagerly. Deeply interested in his suffering companion, he ventured out at midnight, pulled a quantity of grass and the tender shoots of such trees as goats are fond of browsing, and carrying them to her in the cave, had the pleasure of finding that she ate of them ravenously. The goat remained for some time the companion of his solitude, and he had the satisfaction of seeing, or rather knowing, that she was rapidly recovering. At this time it happened that the servant to whom was entrusted the secret of his retreat, fell sick, and it was necessary to send another with provisions. The goat, on this occasion, happening to be near the mouth of the cave, opposed the man's entrance with all her might, butting at him most furiously. The gentleman in the cave, hearing an unusual noise, went forward a few yards, and then receiving the usual watchword from his new attendant, came to the entrance, and, after a few words of interposition, the faithful goat permitted the man to enter. So resolute was the animal on this occasion, that the gentleman felt convinced she would have died in his defence.

